# Androgens in Patients With Luminal B and HER2 Breast Cancer Might Be a Biomarker Promoting Anti-PD-1 Efficacy

**DOI:** 10.3389/fonc.2022.917400

**Published:** 2022-06-29

**Authors:** Peng Li, Wenhui Yuan, Ruan Wu, Chuqian Zeng, Ke Li, Ligong Lu

**Affiliations:** ^1^ Guangdong Provincial Key Laboratory of Tumor Interventional Diagnosis and Treatment, Zhuhai Institute of Translational Medicine, Zhuhai People’s Hospital Affiliated with Jinan University, Jinan University, Zhuhai, China; ^2^ The Biomedical Translational Research Institute, Faculty of Medical Science, Jinan University, Guangzhou, China; ^3^ Anhui Provincial Center for Disease Control and Prevention, Hefei, China; ^4^ The First Affiliated Hospital, Jinan University, Guangzhou, China; ^5^ Department of Infectious Disease, Guangdong Second Provincial General Hospital, Guangzhou, China

**Keywords:** androgens, androgen receptor, breast cancer, immune checkpoint receptors, γδ T cells

## Abstract

Endocrine therapy is considered as an effective strategy for estrogen and progestogen receptor (ER and PR)-positive breast cancer (BRCA) patients, whereas resistance to these agents is the major cause of BRCA mortality in women. Immune checkpoint receptor (ICR) blockade is another approach to treat BRCA, but the response rate of this approach for non-triple-negative breast cancer (non-TNBC) is relatively low. Recently, the androgen receptor (AR) has been identified as a tumor suppressor in ER-positive BRCA; however, the relationship between the levels of androgens and ICRs on T cells in BRCA is unclear. We observed that testosterone and dihydrotestosterone (DHT) in patients with HER2 and Luminal B were significantly lower than those in healthy controls, and the expression of AR has significant correlation with overall survival (OS) advantage for Luminal B patients. Moreover, testosterone and DHT were positively correlated with the PD-1 expression on Vδ1^+^ T cells in HER2 and Luminal B patients. These results suggest a potential approach of combining androgens with PD-1 blockade for treating HER2 and Luminal B breast cancer.

## Introduction

Breast cancer is a global threat to women’s health. There is an estimated 2.26 million new cancer cases and 0.68 million cancer deaths among women worldwide in 2020 (1). According to the expression of human epidermal growth factor receptor 2 (HER2) and hormone receptors (ER and PR), breast cancer is classified into four major molecular subtypes, namely, luminal A (HR+/HER2-), luminal B (HR+/HER2+), HER2+, and triple-negative breast cancer (ER-, PR-, and HER2-negative) (2). Endocrine treatment is used as a conventional strategy for ER- and PR-positive patients with BRCA, whereas resistance to these drugs is the major cause of BRCA mortality (3). The need for alternative strategies has renewed interest in androgen therapy, especially as nearly all ER-positive BRCAs express androgen receptor (AR) (4, 5). The role of AR in ER-positive BRCA is controversial, which restricts implementation of AR-directed treatment. However, a recent study identified AR as a tumor suppressor in ER-positive BRCA and supported AR agonism as the optimal AR-directed treatment strategy, revealing a rational therapeutic opportunity (6).

Androgens exert biological functions through binding and activating the AR (7, 8). The biological actions of androgens, including testosterone and DHT as well as androstenedione, dehydroepiandrosterone (DHEA), and its sulfated form (DHEA-S), are normally mediated through the AR, a ligand-dependent nuclear transcription factor (9). Additionally, AR expression was detected in T-lymphocyte, with the highest expression in cytotoxic T cells (10–13), and androgens are described as suppressors of inflammation and immune function (14). Androgen deprivation therapy (ADT), a standard of care in prostate cancer (15), induces expansion of naïve T cells and enhances T-cell responses (9, 16). Inhibition of AR activity in T cells also promotes checkpoint blockade efficacy (17–19). Together, combination of ADT and targeted ICR treatment may be one of the most powerful therapies for malignant tumors in male patients.

Infiltration of immune cells such as CD4, CD8, and γδ T cells in tumor tissue provides one of the major protections in antitumor immunity (20–22). However, tumor microenvironment drives elevated expression of programmed cell death-1 (PD-1), T-cell immunoglobulin and mucin-domain containing-3 (Tim-3), and T-cell immunoreceptor with Ig and ITIM domains (TIGIT) on T cells (23–25). Blocking ICRs or directly reducing their expression has promising effects in reinvigorating antitumor immunity in a wide variety of tumor types (26, 27). Targeting PD-L1 is another therapeutic approach for BRCA, but the response rate for non-TNBC is relatively low (28, 29). Intra-tumoral γδ T-cell signatures emerged as the most significant favorable prognostic in patient with cancer (30). These cells display an innate-like activity and recognize antigens in a major histocompatibility complex-independent manner *via* surface receptor NKG2D (31, 32). Another study showed that an innate-like Vδ1^+^ γδ T-cell compartment in the human breast is associated with remission in TNBC (33). However, whether the level of androgens in patients with BRCA subtypes correlates with T-cell exhaustion is unknown.

Here, we reported that the levels of testosterone and DHT in patients with HER2 and Luminal B were significantly lower than those of healthy donors. Clinical samples showed that AR has a significant correlation with overall survival (OS) advantage for patients with Luminal B subtypes. Indeed, we observed that patient’s T cells exhibited exhausted phenotypes, characterized by increased expression of PD-1, Tim-3, and TIGIT on CD4^+^, CD8^+^, Vδ2^+^, and Vδ1^+^ T cells. Finally, the serum levels of testosterone and DHT in patients with HER2 and Luminal B were positively correlated with the PD-1 expression on Vδ1^+^ T cells. These results suggest that supplementation of androgens may improve the efficacy of anti-PD-1 therapy in patients with HER2 and Luminal B subtypes.

## Materials and Methods

### Ethics Statement

Isolation of peripheral blood mononuclear cells (PBMCs) and tissues from BRCA and healthy donors was approved by the Institutional Review Board of Jinan University, Guangzhou, China.

### Human Samples

A total of 84 patients with BRCA, including HER2, Luminal A, Luminal B, and TNBC who were diagnosed with BRCA by pathologic examination, were recruited from outpatient clinics of the First Affiliated Hospital of Jinan University. Information regarding the characteristics of the patient cohorts is described in **Table 1**. A total of 35 sex- and age-matched healthy donors were enrolled from the medical examination department at the First Affiliated Hospital of Jinan University. PBMCs were collected from the patients with BRCA and healthy donors through the Ficoll-Paque (GE Healthcare) density gradient centrifugation protocol (34). The fresh serum and PBMCs were stored at −80°C, and tissue samples were fixed in formalin and embedded in paraffin.

**Table 1:** Baseline patient and treatment characteristics.

Baseline patient and Treatment characteristics, No. (%)				
Group	Luminal A	Luminal B	HER2	TNBC
**No. of patients**	21	28	18	17
**Age**				
Median	50.0 (46.5-63.5)	53.0 (46.0-59.8)	52.5 (48.3-58.5)	55.0 (48.0-63.0)
Mean	54.6	52.2	51.4	55.1
**Pathologic stage**				
I	7 (33.3)	6 (21.4)	4 (22.2)	4 (23.5)
II	8 (38.1)	14 (50.0)	6 (33.3)	8 (47.1)
III	3 (14.3)	6 (21.4)	5 (27.8)	3 (17.6)
IV	3 (14.3)	2 (7.2)	3 (16.7)	2 (11.8)
**Receipt of chemotherapy**				
Yes	14 (66.7)	17 (60.7)	14 (77.8)	13 (76.5)
No	7 (33.3)	11 (39.3)	4 (22.2)	4 (23.5)
**Menopausal status**				
Premenopausal	9 (42.9)	11 (39.3)	7 (38.9)	6 (35.3)
Postmenopausal	12 (57.1)	17 (60.7)	11 (61.1)	11 (64.7)

### Flow Cytometry

For surface staining, approximately 1×10^6^ PBMCs were incubated with indicated antibodies at 4°C in the dark. PBMCs were stained with PerCP-conjugated anti-human TCR Vδ2 (BioLegend, 331410), FITC-conjugated anti-human TCR Vδ1 (Miltenyi, 130-100-532), V500-conjugated anti-human CD3 (BD Biosciences, 561416), PerCP-conjugated anti-human CD4 (BioLegend, 317431), PerCP-conjugated anti-human CD8 (BioLegend, 300921), Pacific Blue-conjugated anti-human CD279 (BioLegend, 329915), PE/Cy7-conjugated anti-human TIGIT (BioLegend, 372714), and APC-conjugated anti-human Tim-3 (BioLegend, 345011). After incubation for 20 min, cells were washed with phosphate buffer solution (PBS) and analyzed using the BD FACS Verse Flow Cytometer (BD Biosciences). Flow cytometry data were analyzed with FlowJo software (v.10). The gating strategies are shown in **Figure 2**.

### Immunohistochemistry

Paraffin-embedded HER2, Luminal B, Luminal A, and TNBC specimens were obtained from the First Affiliated Hospital, Jinan University (Guangzhou, China). AR (CST, 5153, 1:500 dilution) staining was performed according to the following standard protocol. Patient tissues were incubated with AR antibody at 4°C overnight. Sections were rinsed with PBS and incubated with goat anti-rabbit antibody for another 1 h. Slides were further developed with diaminobenzidine (DAB) substrate and then counterstained with Mayer’s hematoxylin (34).

### ELISAs

Human serum (healthy donors and patients with BRCA) was obtained as described above. Assays using the ELISA kits for human testosterone (Alpha Diagnostic International, 1880) and dihydrotestosterone (Alpha Diagnostic International, 1940) were performed according to the manuals.

### GEPIA and Kaplan–Meier Plotter

**Figure 1:**
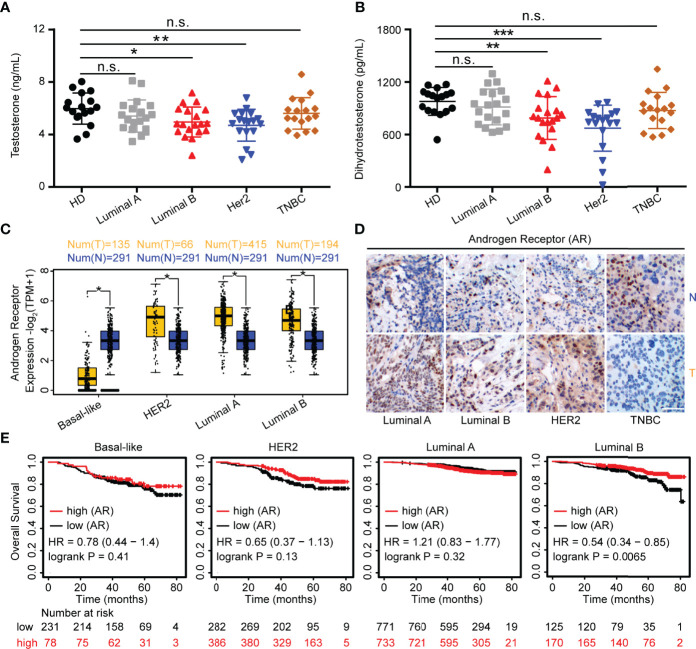
Androgens are reduced in HER2 and Luminal B subjects. **(A, B)** Sera from patients (Luminal A, *n* = 19; Luminal B, *n* = 19; HER2, *n* = 18; TNBC, *n* = 17) and healthy controls (*n* = 17) were used for detection of testosterone and DHT by ELISA. **(C)** Level of AR in breast cancers from the GEPIA dataset (para-cancerous tissues, *n* = 291; cancer tissues with HER2, *n* = 66; Luminal A, *n* = 415; Luminal B, *n* = 194; basal-like/triple negative, *n* = 135). Para-cancerous tissues (blue) are labeled as “normal” and tumor tissues (yellow) are labeled as “tumor”. **(D)** Immunohistochemistry for AR expression in cancer tissues and normal tissues with Luminal A, Luminal B, HER2, and TNBC patients. Scale bar = 50 µm. **(E)** Overall survival of BRCA with high and low AR expression as defined by the median. Analysis of survival data using the Kaplan–Meier plotter online tool. Significance was set to *p* < 0.05 and represented as *p < 0.05, **p < 0.01, ***p < 0.001, and ****p < 0.0001, n.s., not significant (see *Materials and Methods* for statistical tests used).

The RNA-Seq dataset that supports the conclusions of this article is available from Gene Expression Profiling Interactive Analysis (GEPIA) (35). GEPIA is a newly developed interactive platform for elaborating the RNA-Seq data of 8,587 normal and 9,736 tumor samples from the TCGA and the Genotype-tissue Expression dataset, utilizing a standard processing pipeline (36). In this study, box plots showed the expression of signature gene sets in para-cancerous tissues (*n* = 291) and cancer tissues with HER2 (*n* = 66), Luminal A (*n* = 415), Luminal B (*n* = 194), and basal-like/triple negative (*n* = 135). The Log_2_^FC^ Cutoff =1, and the *p*-value cutoff = 0.01. For OS of patients with BRCA, we used the Kaplan–Meier Plotter online tool to evaluate the prognostic value of AR mRNA expression in patients with HER2, Luminal A, Luminal B, and basal-like (37). The OS of patients was analyzed with a 50% (median) cutoff for both low- and high-expression groups. Restrict analysis to PAM subtypes: HER2 (*n* = 295), Luminal A (*n* = 1504), Luminal B (*n* = 668), and basal-like (*n* = 309). Significance was set to *p*-value < 0.05. Information on the number of patients, median values of mRNA expression, hazard ratio (HR), and *p*-value can be found on the Kaplan–Meier Plotter web page and **Figure 1**.

### Statistical Analysis

**Figure 2:**
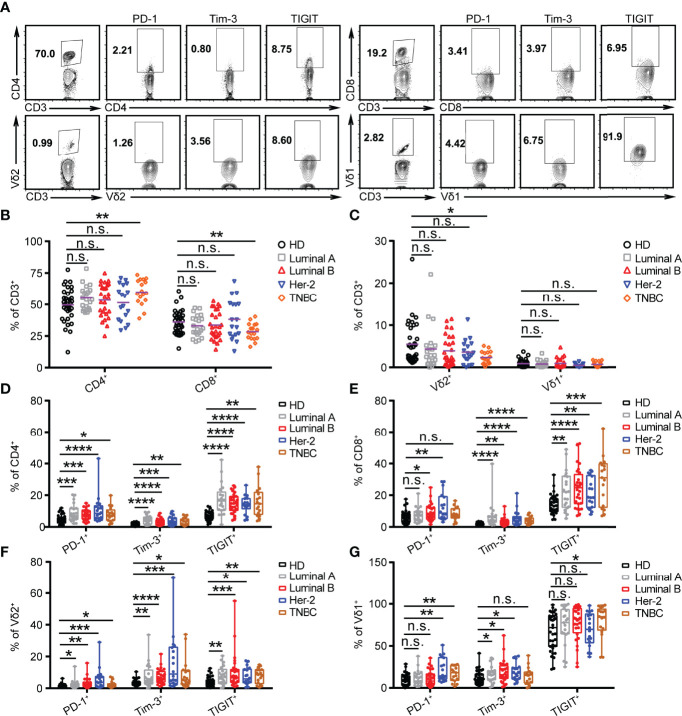
PD-1 is highly expressed on CD4, CD8, and γδ T cells in HER2 cancer patients. **(A)** Representative flow cytometry plots showed the gating strategy. **(B, C)** Summarized percent of CD4^+^, CD8^+^, Vδ2^+^, and Vδ1^+^ CD3 T cells in BRCA subtypes (Luminal A, *n* = 21; Luminal B, *n* = 28; HER2, *n* = 18; TNBC, *n* = 17) and healthy donors (*n* = 35). **(D–G)** Expression of PD-1^+^, Tim-3^+^, and TIGIT^+^ on CD4^+^, CD8^+^, Vδ2^+^, and Vδ1^+^ T cells in healthy donors and BRCA patients. Significance was set to *p* < 0.05 and represented as *p < 0.05, **p < 0.01, ***p < 0.001, and ****p < 0.0001, n.s., not significant (see *Materials and Methods* for statistical tests used).

Statistical analyses were performed using GraphPad Software (v.6.0). For human sample analysis, the data distribution was first checked using a Kolmogorov–Smirnov test. If the data fitted a normal distribution, a two-tailed unpaired Student’s *t*-test was used when variances were similar, whereas a two-tailed unpaired Student’s *t*-test with Welch’s correction was used when variances were different. If the data did not fit a normal distribution, a Mann–Whitney *U* test was used (27). Unpaired Student’s t-test was used in **Figures 1A, C**, **2B–G** (HD vs. Luminal A, CD4^+^CD3^+^, CD8^+^CD3^+^, PD-1^+^CD8^+^, and Tim-3^+^Vδ1^+^; HD vs. Luminal B, PD-1^+^CD4^+^, TIGIT^+^CD4^+^, CD8^+^CD3^+^, and Tim-3^+^Vδ1^+^; HD vs. HER2, CD4^+^CD3^+^, CD8^+^CD3^+^, and Tim-3^+^Vδ1^+^; HD vs. TNBC, CD4^+^CD3^+^, PD-1^+^CD4^+^, TIGIT^+^CD4^+^, CD8^+^CD3^+^, PD-1^+^CD8^+^, and Tim-3^+^Vδ1^+^). Mann–Whitney test was used in **Figures 1B**, **2B–G** (HD vs. Luminal A, PD-1^+^CD4^+^, Tim-3^+^CD4^+^, TIGIT^+^CD4^+^, Tim-3^+^CD8^+^, TIGIT^+^CD8^+^, Vδ2^+^CD3^+^, PD-1^+^Vδ2^+^, Tim-3^+^Vδ2^+^, TIGIT^+^Vδ2^+^, Vδ1^+^CD3^+^, PD-1^+^Vδ1^+^, and TIGIT^+^Vδ1^+^; HD vs. Luminal B, CD4^+^CD3^+^, Tim-3^+^CD4^+^, PD-1^+^CD8^+^, Tim-3^+^CD8^+^, TIGIT^+^CD8^+^, Vδ2^+^CD3^+^, PD-1^+^Vδ2^+^, Tim-3^+^Vδ2^+^, TIGIT^+^Vδ2^+^, Vδ1^+^CD3^+^, PD-1^+^Vδ1^+^, and TIGIT^+^Vδ1^+^; HD vs. HER2, PD-1^+^CD4^+^, Tim-3^+^CD4^+^, TIGIT^+^CD4^+^, PD-1^+^CD8^+^, Tim-3^+^CD8^+^, TIGIT^+^CD8^+^, Vδ2^+^CD3^+^, PD-1^+^Vδ2^+^, Tim-3^+^Vδ2^+^, TIGIT^+^Vδ2^+^, Vδ1^+^CD3^+^, PD-1^+^Vδ1^+^, and TIGIT^+^Vδ1^+^; HD vs. TNBC, Tim-3^+^CD4^+^, Tim-3^+^CD8^+^, TIGIT^+^CD8^+^, Vδ2^+^CD3^+^, PD-1^+^Vδ2^+^, Tim-3^+^Vδ2^+^, TIGIT^+^Vδ2^+^, Vδ1^+^CD3^+^, PD-1^+^Vδ1^+^, and TIGIT^+^Vδ1^+^). Significance was set to *p* < 0.05 and represented as *p < 0.05, **p < 0.01, ***p < 0.001, and ****p < 0.0001, n.s., not significant. Data were presented as mean or mean ± SD.

## Results

### Androgens Are Reduced in HER2 and Luminal B Subjects

Endocrine therapy is applied as a classical method for ER- and PR-positive BRCA patients; however, the potential role of androgens and AR in patients with BRCA subtypes has not been well defined. Thus, we recruited 84 patients with BRCA (Luminal A, Luminal B, HER2, and TNBC), and a summary of the characteristics of patients is described in **Table 1**. To investigate the role of androgens and AR in patients with BRCA, we determined the serum levels of androgens and found that the testosterone and DHT were significantly decreased in human participants with HER2 and Luminal B, while it was barely changed in Luminal A and TNBC patients (**Figures 1A, B**). The mRNA levels of AR were analyzed using the GEPIA online tool. We found that the levels of AR were higher in cancer tissues than that in para-cancerous tissues at Luminal A, Luminal B, and HER2-positive breast cancer, while it was the exact opposite in TNBC (**Figure 1C**). The results of immunohistochemical staining further validated the higher level of AR protein in tumor tissues than in para-cancerous tissues of patients (Luminal A, Luminal B, and HER2) (**Figure 1D**). Moreover, the expression of AR has a significant correlation with OS advantage for patients with Luminal B subtypes, whereas OS in HER2 marginally increased (**Figure 1E**). Collectively, these results strongly indicate that androgens and AR are prognostic factors associated with better patient survival in Luminal B or HER2 subtypes.

### PD-1 Is Highly Expressed on CD4, CD8, and γδ T Cells in HER2 Cancer Patients

Previous studies demonstrate that an innate-like Vδ1^+^ γδ T cell compartment in the human breast is associated with remission in TNBC (33). However, whether the levels of ICRs, including PD-1, Tim-3, and TIGIT, on T cells (αβ and γδ) vary with different BRCA subtypes is unclear. Therefore, the expression of ICRs on CD4, CD8, Vγ9Vδ2 (Vδ2), and Vδ1 T cells from the PBMCs of breast cancer patients was analyzed. The gating strategies are shown in **Figure 2A**. Consistent with our previous reports (34), the percentage of Vδ2^+^CD3^+^ and CD8^+^CD3^+^ T cells in PBMCs of patients with TNBC was significantly lower than that of healthy donors, whereas the proportion of CD4^+^CD3^+^ T cells was significantly increased in TNBC (**Figures 2B, C**). Meanwhile, an immune exhaustion phenotype was verified in BRCA as indicated by increased expression of PD-1, TIGIT, and Tim-3 on CD4, CD8, Vδ2, and Vδ1 T cells, especially the percentage of PD-1^+^CD4^+^, PD-1^+^CD8^+^, PD-1^+^Vδ1^+^, and PD-1^+^Vδ2^+^ T cells in patients with HER2 (**Figures 2D–G**). These data suggest that the use of the anti-PD-1 inhibitor to target PD-1^+^ on T cells should be considered as a cancer immunotherapy for patients with HER2 subtypes.

### The Levels of Testosterone and DHT in HER2 and Luminal B Patients Were Positively Correlated With the PD-1 Expression on Vδ1^+^ T Cells

**Figure 3:**
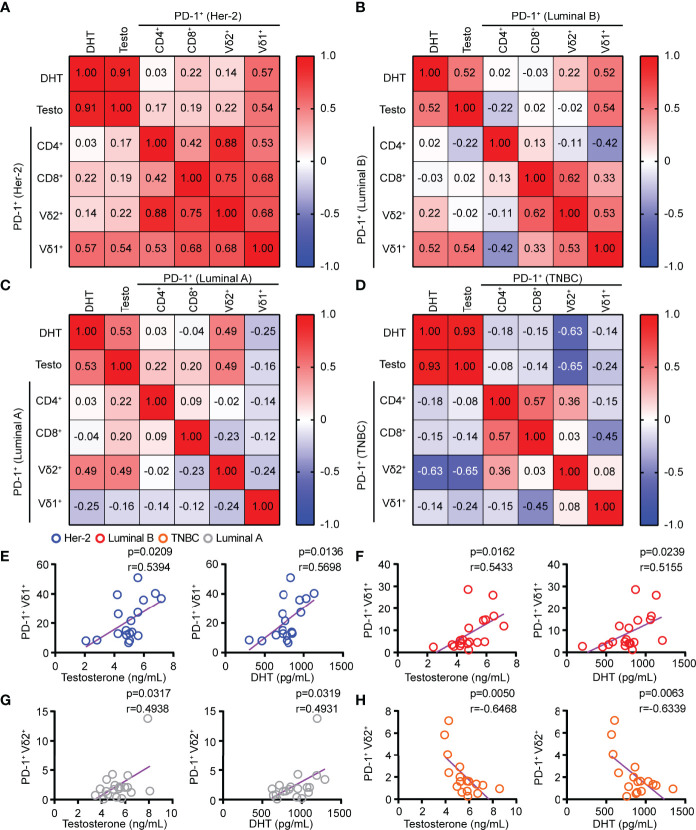
The levels of testosterone and DHT in HER2 and Luminal B patients were positively correlated with the PD-1 expression on Vδ1^+^ T cells. **(A–D)** Linear regression analysis between androgen (testosterone and DHT) levels and PD-1^+^ on T cells (CD4, CD8, Vδ2, and Vδ1) in patients with HER2 (*n* = 18), Luminal B (*n* = 19), Luminal A (*n* = 19), and TNBC (*n* = 17). **(E–H)** Correlation between serum androgen levels and PD-1^+^ on γδ T cells (Vδ2^+^ and Vδ1^+^) in patients with BRCA (HER2, *n* = 18; Luminal B, *n* = 19; Luminal A, *n* = 19; TNBC, *n* = 17). Two-tailed Pearson correlation **(A–H)**.

Blockade of PD-1 expression on T cells *via* anti-PD-1 monoclonal antibody has shown great promise for successful cancer treatment by overcoming T-cell exhaustion. However, the response rates of anti-PD-1 treatment were determined by the abundance of ICRs on T cells. Our results demonstrated that the serum levels of testosterone and DHT in HER2 and Luminal B patients had a significant positive correlation with PD-1^+^ on Vδ1^+^ T cells but not PD-1^+^ on CD4^+^ and CD8^+^ T cells (**Figures 3A–F**). Furthermore, the levels of testosterone and DHT were positively correlated with PD-1^+^ on Vδ2^+^ T cells in patients with Luminal A (**Figure 3G**), but inversely correlated with PD-1^+^ on Vδ2^+^ T cells in TNBC (**Figure 3H**). In summary, these results indicate that the combination of the anti-PD-1 inhibitor and androgens may have a unique therapeutic potential in treating HER2 and Luminal B cancer through Vδ1^+^ T cells.

## Discussion

Herein, we provide extensive evidence that patients with HER2 and Luminal B might benefit from androgens combined with ICR-targeted therapies. Indeed, a recent study clarified that the AR agonist/AR signaling pathway has a tumor suppressor role in ER-positive BRCA (6). Therefore, this insightful therapy strategy has the potential to become an alternative endocrine treatment for BRCA, especially for those resistant to current forms of endocrine therapy. In this study, we reported the levels of androgens (testosterone and DHT) and ICRs of T cells originated from BRCA subtypes, and found that androgens were significantly decreased in HER2 and Luminal B patients compared with healthy controls. Interestingly, in an attempt to find a correlation of immune exhaustion with women’s diseases, we performed an analysis that provided clinical data for a correlation between androgens and ICRs, and showed that testosterone and DHT were significantly positively correlated with PD-1^+^Vδ1^+^ T cells in HER2 and Luminal B.

Recently, in a clinical trial on patients with metastatic, hormone-sensitive prostate cancer, the OS was significantly longer with a therapy combining darolutamide, ADT, and docetaxel than with placebo plus ADT and docetaxel, and the addition of darolutamide led to improvement in key secondary end points (38). Xiangnan et al. demonstrated that AR blockade sensitizes tumor-bearing hosts to effective checkpoint blockade by directly enhancing CD8 T-cell function (17). Studies by Terrisse et al. also show that both immune system and intestinal microbiota determine efficacy of ADT against prostate cancer (39). These data indicate that male patients can benefit from ADT. However, female sex has been suggested as a negative predictive factor for response of melanoma patients to ani-PD-1 therapy (40). One explanation for this phenomenon might be the paucity of partially exhausted PD-1-positive CD8 T cells associated with response to combined ICR inhibition in women (41).

A previous work revealed that a large population of Vδ1^+^ T cells in human breast tumors, and the progression-free survival and OS were correlated with the proportion of Vδ1^+^ T cells, but not with either total γδ T cells or Vδ2^+^ T cells (33). It is well known that PD-L1 expressed on tumor cells directly binds PD-1-positive T cells to reduce their effector function and induce exhaustion, which leads to tumor immune evasion (20, 23). Interestingly, the levels of androgens and ICRs on T cells in BRCA subtypes including HER2, Luminal A, Luminal B, and TNBC have not been characterized. Our results showed that PD-1 was highly expressed on CD4, CD8, Vδ2, and Vδ1 T cells in HER2 subtype patients. In addition, the precise effects of androgens/AR pathway on the expression of ICRs on T cells and the underlying mechanisms remain to be further investigated.

In summary, our preliminary evidence indicated that the combination of androgens and anti-PD-1 inhibitor targeted therapy might be a new and effective approach to improve antitumor response of HER2 and Luminal B patients through Vδ1^+^ T cells.

## Data Availability Statement

The original contributions presented in the study are included in the article/supplementary material. Further inquiries can be directed to the corresponding authors.

## Ethics Statement

The studies involving human participants were reviewed and approved by the Institutional Review Board of Jinan University, Guangzhou, China. Written informed consent for participation was not required for this study in accordance with the national legislation and the institutional requirements.

## Author Contributions

PL, WY, and KL conceived the project and wrote the manuscript. PL, WY, and RW performed research and made the figures. CZ and LL provided technical assistance. PL, WY, and KL contributed to manuscript preparation. All authors contributed to the article and approved the submitted version.

## Funding

This work was supported by the Guangdong Second Provincial General Hospital (grant TJGC-2021020 to KL) and the Medical Scientific Research Foundation of Guangdong Province, China (grant A2022417 to KL).

## Conflict of Interest

The authors declare that the research was conducted in the absence of any commercial or financial relationships that could be construed as a potential conflict of interest.

## Publisher’s Note

All claims expressed in this article are solely those of the authors and do not necessarily represent those of their affiliated organizations, or those of the publisher, the editors and the reviewers. Any product that may be evaluated in this article, or claim that may be made by its manufacturer, is not guaranteed or endorsed by the publisher.
